# Heterogeneous Disease Trajectories Explain Variable Radiographic, Function and Quality of Life Outcomes in the Canadian Early Arthritis Cohort (CATCH)

**DOI:** 10.1371/journal.pone.0135327

**Published:** 2015-08-24

**Authors:** Cheryl Barnabe, Ye Sun, Gilles Boire, Carol A. Hitchon, Boulos Haraoui, J. Carter Thorne, Diane Tin, Désirée van der Heijde, Jeffrey R. Curtis, Shahin Jamal, Janet E. Pope, Edward C. Keystone, Susan Bartlett, Vivian P. Bykerk, CATCH Investigators

**Affiliations:** 1 Department of Medicine and Department of Community Health Sciences, University of Calgary, Calgary, Alberta, Canada; 2 Division of Rheumatology, Mount Sinai Hospital, University of Toronto, Toronto, Ontario, Canada; 3 Department of Medicine, Universite de Sherbrooke, Sherbrooke, Quebec, Canada; 4 Department of Medicine, University of Manitoba, Winnipeg, Manitoba, Canada; 5 Rheumatic Disease Unit, Institut de Rheumatologie, Montreal, Quebec, Canada; 6 Southlake Regional Health Centre, Newmarket, Ontario, Canada; 7 Leiden University Medical Centre, Leiden, The Netherlands; 8 University of Alabama at Birmingham, Birmingham, Alabama, United States of America; 9 University of British Columbia, Vancouver, British Columbia, Canada; 10 Western University, London, Ontario, Canada; 11 McGill University, Montreal, Quebec, Canada; 12 Hospital for Special Surgery, Weill Cornell Medical College, New York, New York, United States of America; Nippon Medical School Graduate School of Medicine, JAPAN

## Abstract

Our objective was to identify distinct trajectories of disease activity state (DAS) and assess variation in radiographic progression, function and quality of life over the first two years of early rheumatoid arthritis (ERA). The CATCH (Canadian early ArThritis CoHort) is a prospective study recruiting ERA patients from academic and community rheumatology clinics in Canada. Sequential DAS28 scores were used to identify five mutually exclusive groups in the cohort (n = 1,586) using growth-based trajectory modeling. Distinguishing baseline sociodemographic and disease variables, treatment required, and differences in radiographic progression and quality of life measures over two years were assessed. The trajectory groups are characterized as: Group 1 (20%) initial high DAS improving rapidly to remission (REM); Group 2 (21%) initial moderate DAS improving rapidly to REM; Group 3 (30%) initial moderate DAS improving gradually to low DAS; Group 4 (19%) initial high DAS improving continuously to low DAS; and Group 5 (10%) initial high DAS improving gradually only to moderate DAS. Groups differed significantly in age, sex, race, education, employment, income and presence of comorbidities. Group 5 had persistent steroid requirements and the highest biologic therapy use. Group 2 had lower odds (OR 0.22, 95%CI 0.09 to 0.58) and Group 4 higher odds (OR 1.94, 95%CI 0.90 to 4.20) of radiographic progression compared to Group 1. Group 1 had the best improvement in physical function (Health Assessment Questionnaire 1.08 (SD 0.68) units), Physical Component Score (16.4 (SD 10.2) units), Mental Component Score (9.7 (SD 12.5) units) and fatigue (4.1 (SD 3.3) units). In conclusion, distinct disease activity state trajectories explain variable outcomes in ERA. Early prediction of disease course to tailor therapy and addressing social determinants of health could optimize outcomes.

## Introduction

High variability has been noted within and between individuals with early rheumatoid arthritis (ERA), not only in their initial presentation, but also in the clinical progression of their disease [1]. This suggests the possibility that discrete disease trajectories exist at presentation, which may be explained by personal variables including sociodemographic status, disease characteristics, and/or health status, but also treatment received including initial treatment choice and dosing, escalation schemes, timing of therapy initiation, use of steroids, and tight control algorithms [2]. Understanding which baseline characteristics influence patient trajectories and important outcomes such as radiographic progression, physical function and quality of life measures in ERA could provide additional prognostic information to inform treatment strategy.

Recently, an ERA cohort study reported differing trajectories based on patient DAS28 scores over one year, showing patients could have a fast or slow response or a poor outcome, despite a treat-to-target strategy, determined with growth mixture modelling [3]. Analogous to studies defining ‘responders’ and ‘non-responders’, this analytic strategy allows further refinement of outcome ascertainment, as homogeneous groups of patients similar to each other at baseline and over time for a given outcome can be characterized longitudinally in a dataset, with the important predictors of the various treatment courses determined. Building on this important work, we applied a group-based trajectory modelling strategy to data from the CATCH (Canadian Early Arthritis Cohort) Study. Our objectives were to i) evaluate whether patients could be clustered over time based on their disease activity; ii) identify whether group-based modifiable and non-modifiable sociodemographic and disease related factors differentiate group membership at disease onset; and iii) determine if such clinical distinctions are concordant with differences in radiographic progression, physical function and quality of life measures during the first two years of disease.

## Methods

### Subjects

Data from the CATCH study (January 2007 to May 2014) were used for this study. CATCH is a prospective observational cohort study of adults with ERA from 21 academic and community-based clinics across Canada that has been previously described [4]. Briefly, patients were included with ≤12 months of symptoms, ≥2 swollen joints or 1 swollen metacarpophalangeal (MCP) or proximal interphalangeal (PIP) joint, plus at least one RA related disease factor. The primary analysis included all CATCH subjects observed in at least 2 study visits and meeting either 2010 [5] or 1987 [6] RA Classification Criteria at the baseline visit.

### Data Elements

Standardized study visits include a baseline assessment at the time of enrolment, quarterly reviews in the first year, biannually in year 2, and annually thereafter. As this is an observational study performed in clinical settings, not all subjects have anti-cyclic citrullinated peptide (anti-CCP) tested (as it is not reimbursed by all local health authorities). Physician measured joint counts, assessment of disease activity, medication received in a treat-to-target strategy, C-Reactive Protein (CRP) and Erythrocyte Sedimentation Rate (ESR), are collected at all visits. Patient self-reported function (Health Assessment Questionnaire, HAQ [7]) and visual analogue scales for global evaluation of well-being and pain in the past week (rated from 0–10) are collected every 3 months in the first year, and every 6 months in the second year. Subjects are classified for their disease activity state at each visit based on the Disease Activity Score using 28 joints (DAS28-ESR) with established cutpoints: remission (REM, DAS28<2.6), low disease activity state (LDAS, DAS28 2.6-≤3.2), moderate disease activity state (MDAS, DAS28 3.2–5.1) or high disease activity state (HDAS, DAS28 >5.1) [8].

### Radiographic Measures

Sequential-order van der Heijde modification of Sharp scores (vdHSS) were assigned by one trained reader (intra-class correlation coefficient for intra-observer variability 0.74) in a subset of patients (n = 488, 31%) who had annual serial radiographs performed and at least 2 study visits [9]. Significant radiographic progression was defined as an annual increase above the smallest detectible change (SDC), which was 3.5 units/year in this cohort [10]. Radiographs performed 3–6 months prior to study entry were not repeated but were used as baseline films.

### Quality of Life Measures

The Veteran’s RAND 12 Item Health Survey (VR-12) [11] are completed annually. From twelve items in the questionnaire, a Physical Health Summary Measure (PCS-physical component score) and a Mental Health Summary Measure (MCS-mental component score) are summarized. The minimum clinically important difference (MCID) for these scores is ≥2.5 [12]. Fatigue was also considered as a quality of life measure in this study, captured by a 0–10 numeric rating scale, with an MCID of 2 [13].

### Statistical Methods

The group-based trajectory modelling (GBTM) strategy proposed by Nagin [14] was applied to the data to test for the existence of, and optimal number of, distinct groups for analysis. In the first step, the Bayesian information criterion (BIC) statistic [15] is calculated to provide guidance on the number of distinct trajectory groups that can be defined from the data. In the second stage of the process, a cubic polynomial is used to specify the shape of each trajectory. Clinical acumen is applied to summarize the distinctive features of the data in as parsimonious a fashion as possible. Subjects are then assigned to the group where their posterior membership probability was highest (i.e. to which they most likely belong). The analysis was carried out in SAS (version 9.2), using PROC TRAJ, which uses a general quasi-Newton procedure to estimate parameters that maximize the likelihood function.

Descriptive statistics for continuous variables are presented as means (standard deviation, SD) and categorical variables as frequencies with percentages. Sociodemographic, disease and treatment variables distinguishing the groups were identified by chi-square tests for categorical variables and by Kruskal–Wallis one-way ANOVA for continuous variables. We adjusted for multiple comparisons using the Bonferroni correction.

Due to the limitations of radiographic data analysis using last-observation-carried-forward or linear imputation we used the multiple imputation method described by Baron [16].

### Ethics

Individual consent was obtained, following ethics approval through each site’s Research Ethics Board.

## Results

### Subject Cohort

**Fig 1:**
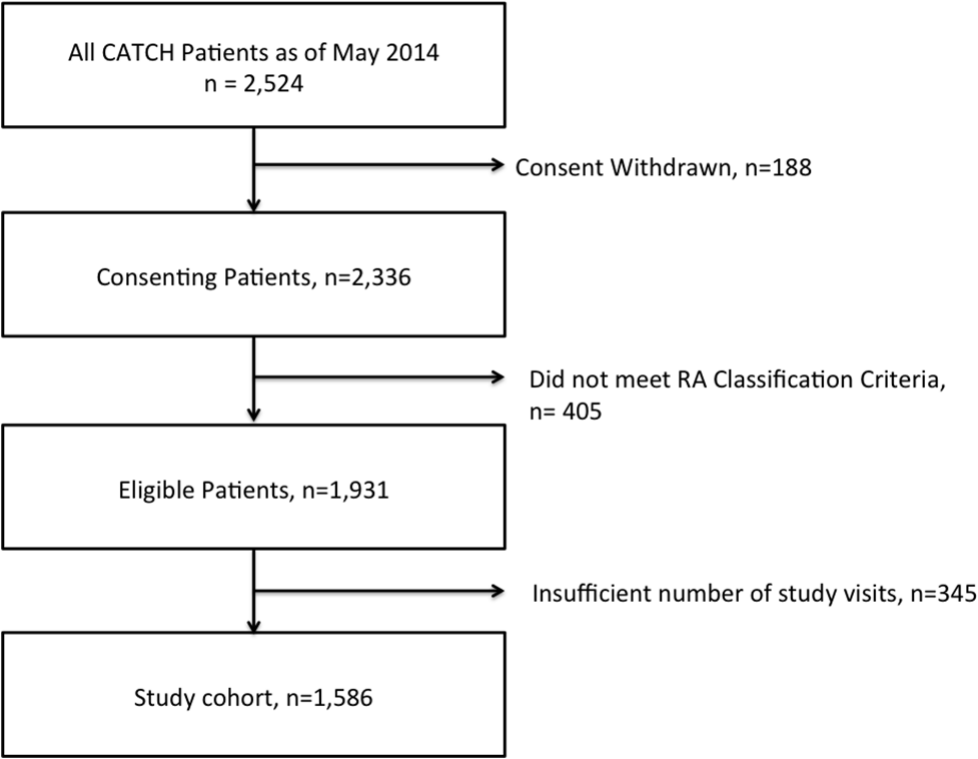
Cohort Derivation. Flow diagram of subjects included in the study.

As of May 2014, a total of 2,524 subjects were enrolled in CATCH. Of these, 1,586 subjects met RA classification criteria, consented to ongoing study participation, and had a minimum of 2 study visits over 24 months (Fig 1).

### Subject baseline characteristics

The majority were females (n = 1152, 73%) with a mean age of 53.5 (SD 14.7) years; 70% (n = 1104) were either rheumatoid factor (RF) or anti-CCP positive (Table 1). Consistent with Canadian population demographics, 82% (n = 1305) of the cohort was Caucasian. Nearly 1 in 5 (18%, n = 290) were current smokers. Participants entered the cohort with a mean of 181 (SD 103) days of symptoms, 7.8 (SD 6.1) swollen joints (28 joint count), a DAS28 of 5.06 (SD 1.45) and a HAQ of 1.03 (SD 0.71). At the baseline visit, 508 subjects (32%) were started on methotrexate monotherapy (>15 mg weekly), 685 (43%) on methotrexate combined with another DMARD, and 253 (16%) on non-methotrexate DMARDs. At the baseline visit, 482 subjects (30%) were receiving oral and 354 (22%) parenteral corticosteroid therapy. Radiographic datasets were available for 488 subjects, who did not differ systematically from the total population under study (Table 1).

**Table 1:** Inception characteristics of subjects in CATCH and of patients with available serial radiographs. Results are reported as a Mean (SD) unless otherwise noted. RF, rheumatoid factor; Anti-CCP, anti-cyclic citrullinated peptide; ESR, erythrocyte sedimentation rate; DAS28, disease activity score using a 28-joint count; HAQ Health Assessment Questionnaire; DMARD, disease modifying anti-rheumatic drug

	All Subjects (n = 1586)	Patients with radiographic data* (n = 488)
Age, years	53.5 (14.7)	54.0 (14.5)
Age >50 years, n (%)	961 (61%)	300 (62%)
Female, n (%)	1152 (73%)	359 (74%)
Number of comorbidities**	2.1 (2.0)	1.7 (1.8)
Current smoker, n (%)	290 (18%)	82 (17%)
Caucasian, n (%)	1305 (82%)	402 (83%)
Education After High School	814 (52%)	251 (52%)
Household Income >$50,000 Canadian/annum	450 (43%)	133 (42%)
Full time employment, n(%)	876 (55%)	254 (52%)
Living Alone, n(%)	397 (25%)	120 (25%)
RF positive, n (%)	955/1457 (66%)	302/474 (64%)
Anti-CCP positive, n (%)	703/1108 (63%)	272/375 (73%)
Patients with erosions***, n (%)	352/1306 (27%)	142/475 (30%)
Tender Joint Count (/28)	8.7 (6.6)	8.7 (7.0)
Swollen Joint Count (/28)	7.8 (6.1)	8.6 (6.3)
ESR (mm/h)	27.7 (23.0)	28.0 (23.5)
Patient Global Score (0–10)	5.8 (2.9)	5.9 (3.0)
Physician Global Score (0–10)	4.9 (2.5)	4.9 (2.6)
Pain Score (0–10)	5.5 (2.9)	5.6 (2.8)
DAS28	5.06 (1.45)	5.09 (1.54)
HAQ (0–3)	1.03 (0.71)	1.04 (0.70)
Symptom duration in days	181 (103)	180 (99)
**Therapy at baseline visit**		
Methotrexate monotherapy	508 (32%)	157 (32%)
Non-biologic DMARD combination therapy	685 (43%)	235 (48%)
Non-methotrexate disease modifying therapy	253 (16%)	64 (13%)
Biologic therapy	35 (2%)	11 (2%)
Steroid exposure, oral or parenteral	836 (53%)	284 (58%)

* No significant differences identified except for anti-CCP (p<0.001); Swollen Joint Count (p<0.001), non-biologic DMARD combination therapy (p<0.01) and Steroid exposure (p = 0.003).

** Comorbidities recorded include: angina/heart attack, asthma, other heart problems, hypertension, cerebrovascular disease/accidents, anemia, bronchitis/emphysema, hypercholesterolemia, bowel disease, stomach ulcer, liver disease, kidney disease, tuberculosis, cancer, psoriasis, thyroid disease, diabetes, hepatitis, chronic infection, osteoarthritis, lupus, osteoporosis, back/spine problems, fibromyalgia, fractures, depression, mental illness, alcoholism, severe allergies, thromboembolic disease, Parkinson disease, migraines, seizures/epilepsy, gynecologic/prostate problems, HIV, herpes and/or cold sores.

*** Reported by site investigator; not from central reader

### Trajectory Groups

**Fig 2:**
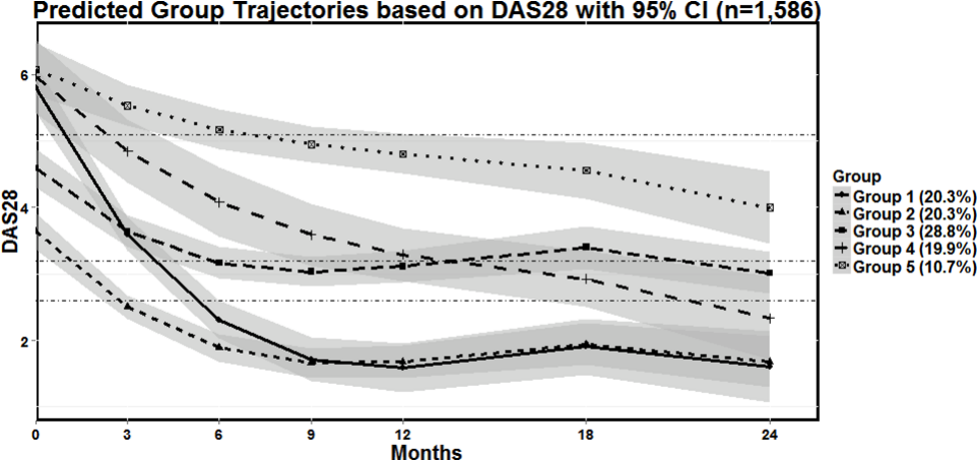
Predicted Group Trajectories in Early Rheumatoid Arthritis based on DAS28 with 95% CI (n = 1,586). Five predicted group trajectories (solid or dashed lines) and 95% confidence interval limits (shaded) are depicted from the group-based trajectory modelling. Percentages reflect the predicted proportion of subjects in each group, which differs marginally from the actual group characterization in the dataset.

Five unique trajectory groups characterized by sequential DAS28 scores were predicted (Fig 2). Subjects were assigned to the predicted groups as follows: Group 1 (20%) initial high disease activity state (HDAS) improving rapidly to remission (REM); Group 2 (21%) initial moderate disease activity state (MDAS) improving rapidly to REM; Group 3 (30%) initial MDAS improving gradually to low disease activity state (LDAS); Group 4 (19%) initial HDAS improving continuously to LDAS; and Group 5 (10%) initial HDAS improving gradually only to MDAS.

### Trajectory Group Characteristics

Table 2 summarizes the sociodemographic, health status and disease characteristics of subjects in the five identified groups. Of the sociodemographic variables, age, race, education, income, and employment status were significantly different between groups (all between-groups adjusted p-values <0.001). A higher proportion of subjects in Groups 4 or 5 were >50 years of age (n = 224, 74% and n = 108, 68% respectively) relative to Groups 1, 2 or 3, and 28% (n = 44) of Group 5 were non-Caucasian. Subjects in Groups 4 or 5 had less frequently achieved higher than high school education (n = 119, 39% and n = 65, 41%, compared to >52% in Groups 1, 2 or 3). In Groups 4 or 5 <44% of subjects were employed compared to >55% in the other groups. With regards to health status, subjects in Groups 4 or 5 had a higher mean number of comorbidities (2.6 (SD 2.1) each, p<0.001). Despite Group 5 having the longest symptom duration at diagnosis (mean 203 days (SD 111)), no differences in disease characteristics at baseline for serology, baseline radiographic scores or the presence of erosions were seen.

**Table 2:** Baseline Sociodemographic, Health Status and Disease Characteristics of the Five Trajectory Groups. Results are reported as a Mean (SD) or n (%), as appropriate. HDAS, high disease activity state (DAS28 > 5.1); REM, remission (DAS28 <2.6); MDAS, moderate disease activity state (DAS28 3.2–5.1); LDAS, low disease activity state (DAS28 2.6–3.2); RF, rheumatoid factor; Anti-CCP, anti-cyclic citrullinated peptide; ESR erythrocyte sedimentation rate; vdHSS van der Heijde Sharp Score

Group	1	2	3	4	5	
	HDAS to REM	MDAS to REM	MDAS to LDAS	HDAS to LDAS	HDAS to MDAS	Adjusted p-value*
**N (%)**	**319 (20%)**	**325 (21%)**	**477 (30%)**	**306 (19%)**	**159 (10%)**	
Age, years	52.0 (15.6)	48.3 (13.9)	54.4 (14.2)	58.6 (13.5)	55.0 (14.0)	<0.001
Age >50 years	179 (56%)	151 (47%)	299 (63%)	224 (74%)	108 (68%)	<0.001
Female	218 (69%)	217 (67%)	377 (79%)	217 (71%)	123 (77%)	0.016
Caucasian	265 (83%)	293 (90%)	373 (78%)	259 (85%)	115 (72%)	<0.001
Education After High School	178 (56%)	206 (64%)	246 (52%)	119 (39%)	65 (41%)	<0.001
Income >$50,000 (Cdn)	106 (48%)	133 (55%)	132 (44%)	55 (32%)	24 (22%)	<0.001
Full time employment	180 (57%)	237 (73%)	260 (55%)	135 (44%)	64 (40%)	<0.001
Living Alone	76 (24%)	69 (21%)	113 (24%)	86 (28%)	53 (33%)	1.0
Number of comorbidities	1.6 (1.6)	1.5 (1.7)	2.2 (2.0)	2.6 (2.1)	2.6 (2.1)	<0.001
Current smoker	43 (14%)	55 (17%)	96 (20%)	58 (19%)	38 (24%)	1.0
Symptom Duration, days	156 (91)	193 (105)	190 (107)	170 (93)	203 (111)	<0.001
RF positive	185 (62%)	203 (67%)	300 (69%)	167 (59%)	100 (73%)	0.8
Anti-CCP positive	138 (65%)	153 (64%)	211 (63%)	124 (60%)	77 (68%)	1.0
DAS28 Score	6.11 (0.87)	3.39 (0.99)	4.44 (0.88)	6.18 (0.90)	5.96 (1.15)	<0.001
Tender Joint Count (/28)	12.4 (6.5)	4.0 (4.1)	6.0 (4.8)	12.4 (6.2)	11.7 (7.1)	<0.001
Swollen Joint Count (/28)	11.1 (6.2)	4.5 (4.6)	5.3 (4.4)	10.6 (5.9)	9.9 (6.6)	<0.001
ESR (mm/h)	34.6 (24.1)	12.0 (12.0)	22.5 (17.9)	40.1 (24.9)	37.0 (24.0)	<0.001
CRP (mg/L)	20.7 (20.1)	7.4 (13.3)	10.1 (13.6)	22.1 (21.0)	17.2 (20.4)	<0.001
Physician Global Score (0–10)	6.2 (2.2)	3.5 (2.2)	4.1 (2.2)	5.7 (2.3)	5.9 (2.3)	<0.001
vdHSS Score**	5.2 (7.3)	3.3 (5.5)	7.7 (11.6)	6.9 (9.1)	4.9 (7.3)	0.3
vdHSS erosion score >1**	37 (36%)	35 (31%)	54 (41%)	38 (42%)	14 (29%)	1.0
Patient Global Score (0–10)	7.0 (2.5)	3.9 (2.7)	5.1 (2.9)	7.1 (2.4)	6.9 (2.5)	<0.001
Pain Score (0–10)	6.6 (2.5)	3.7 (2.7)	4.8 (2.7)	6.8 (2.4)	6.8 (2.4)	<0.001
HAQ Score (0–3)	1.21 (0.70)	0.54 (0.53)	0.83 (0.62)	1.39 (0.65)	1.34 (0.64)	<0.001
Fatigue	6.0 (2.9)	3.8 (2.7)	4.6 (2.9)	6.3 (2.8)	6.5 (2.7)	<0.001
Physical Component Score	35.1 (9.4)	43.1 (10.1)	38.9 (9.9)	31.3 (8.7)	31.9 (9.3)	<0.001
Mental Component Score	44.6 (12.1)	49.0 (10.4)	47.3 (11.2)	42.7 (11.3)	41.5 (12.1)	<0.001
Baseline Visit Treatment Naïve or Minimally Exposed ***	78 (25%)	88 (27%)	104 (22%)	51 (17%)	22 (14%)	0.07
Baseline Visit Methotrexate Monotherapy	111 (35%)	90 (28%)	143 (30%)	116 (38%)	48 (30%)	1.0
Baseline Visit Methotrexate, Dose, mg	21.3 (4.0)	20.7 (3.7)	20.0 (4.2)	20.1 (4.5)	19.2 (4.1)	<0.001
Baseline Visit Combination Therapy Including Methotrexate	137 (43%)	129 (40%)	141 (30%)	111 (36%)	53 (33%)	0.06
Baseline Visit Steroid	181 (57%)	139 (43%)	238 (50%)	185 (61%)	93 (59%)	<0.001
Baseline Visit Biologic	11 (3%)	6 (2%)	4 (1%)	8 (3%)	6 (4%)	1.0

* Bonferroni Method. Table does not display some variables included in the Bonferroni correction, including site size the patient was recruited from, the proportion meeting ACR 1987 or 2010 criteria, all methotrexate exposed (yes/no), the proportion on non-methotrexate DMARDs, and oral steroids. No significant differences were seen in these variables between groups.

** On proportion of subjects with radiographs available: Group 1 n = 102, Group 2 n = 114, Group 3 n = 131, Group 4 n = 91, Group 5 n = 49

*** Not receiving DMARDs or steroids at baseline visit, OR receiving <4 weeks of methotrexate at <15 mg/week

### Treatment by Group

**Fig 3:**
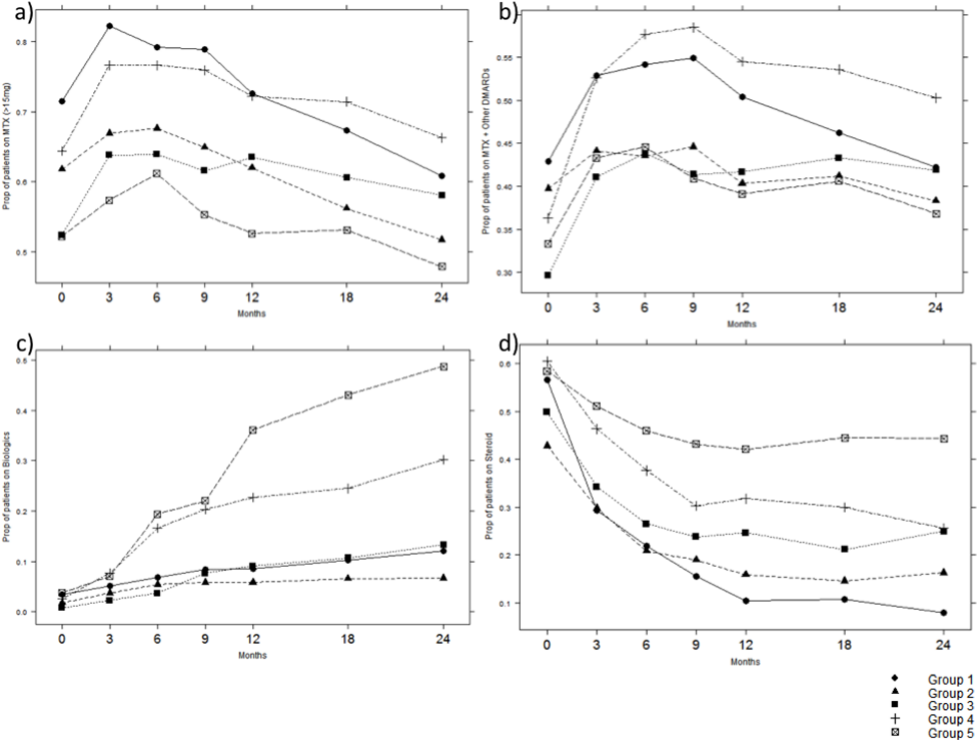
Treatment by Trajectory Group. (A) Group Proportion on Methotrexate (≥15 mg), by Visit Month. (B) Group Proportion on Combination Methotrexate and DMARD Therapy, by Visit Month. (C) Group Proportion on Biologics, by Visit Month. (D) Group Proportion on Corticosteroids, by Visit Month.

The proportion of patients in each group receiving methotrexate, methotrexate in combination with another DMARD, biologics and corticosteroids at each study visit appears in Table 2 and Fig 3. The majority of subjects were already receiving therapy at their baseline visit, with only 14% of Group 5 patients being treatment-naïve, and with >70% of subjects on methotrexate-based regimes (Group 1 82%, Group 2 73%, Group 3 67%, Group 4 85%, Group 5 71%) in a mean dose exceeding 19 mg weekly. Differences emerge during the course of observation. In particular, Group 5 has the lowest proportion of patients remaining on methotrexate and/or combination therapy, but with the highest proportion (nearly 50%) moving on to biologic therapy, yet only achieving MDAS by two years. Group 4, which has the highest proportion of subjects on methotrexate and/or combination therapy, has approximately 30% on biologic therapy by two years, and achieves LDAS by two years. Both these groups also have the highest proportion of subjects on corticosteroids throughout the observation period. In contrast, Group 2 begins in MDAS and achieves REM, has declining methotrexate use over two years of follow-up and <7% prevalence of biologics throughout.

### Radiographic Outcomes

**Fig 4:**
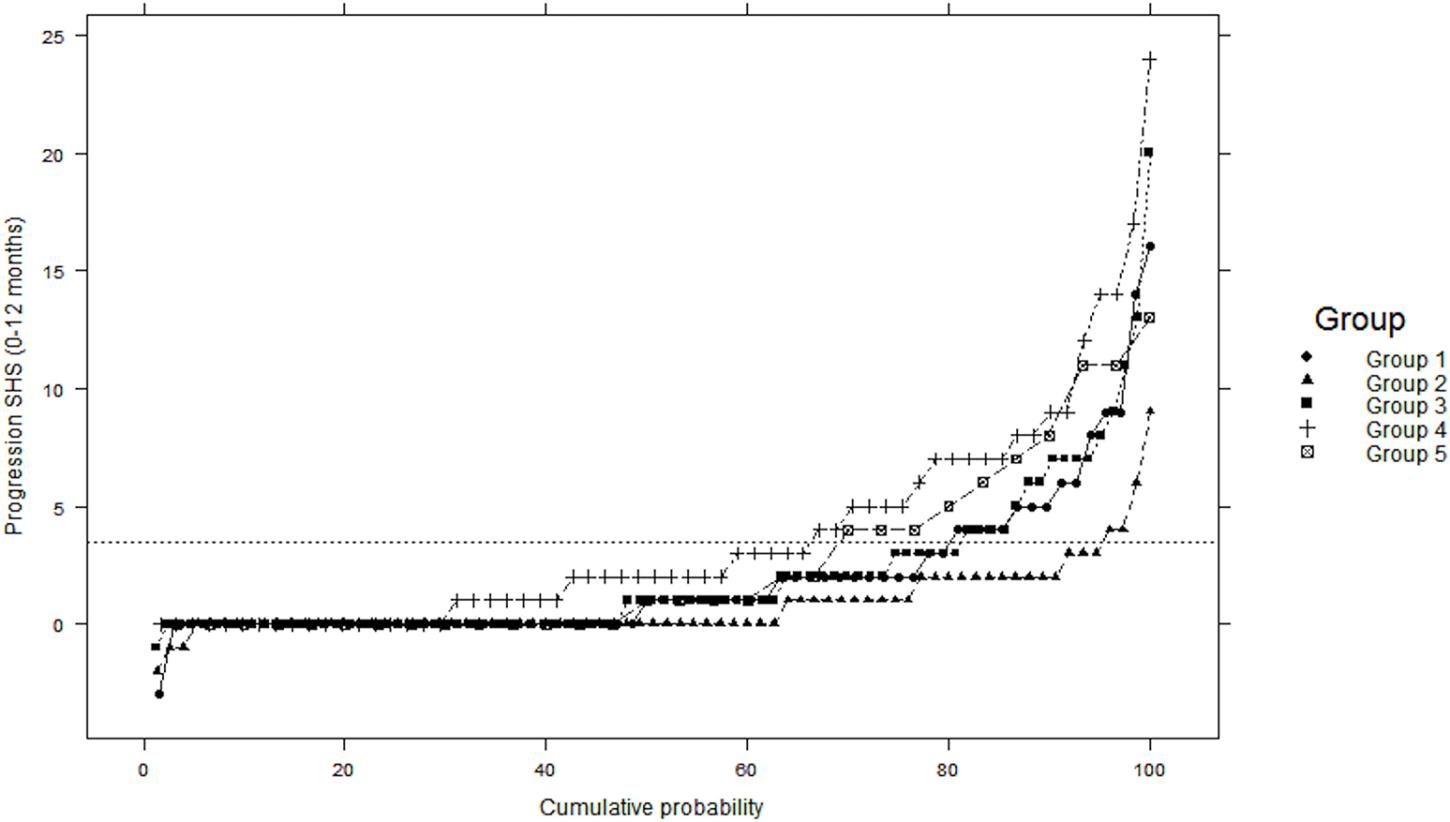
Significant Radiographic Progression During First Year of Follow-up, by Trajectory Group.

There were no significant differences between groups at baseline in the proportion with erosions or vdHSS (Table 2). However, significant differences in radiographic damage progression are evident by the first year of follow-up (between-group chi-squared p<0.001; Fig 4). Radiographic progression was most frequent in subjects assigned to Groups 4 and 5 at 34% (n = 21/61) and 33% (n = 10/30) respectively, compared to 5% (n = 4/11) in Group 2, 19% (n = 16/85) in Group 3 and 21% (n = 14/68) in Group 1. Using Group 1 as the reference in unadjusted logistic regression models, subjects in Group 2 were found to be protected against radiographic progression (OR 0.22, 95%CI 0.09 to 0.58, p = 0.002), and Group 4 was twice as likely to have significant radiographic progression (OR 2.43, 95%CI 1.27 to 4.65, p = 0.007), and with Group 5 demonstrating numerically higher odds of radiographic progression (OR 1.93, 95%CI 0.90 to 4.20, p = 0.09).

### Physical Function

Baseline values by trajectory group are reported in Table 2. Groups 1, 4 and 5 have the worst baseline HAQ score. However, Group 1 has the greatest improvement in HAQ score (mean change of 1.08 (SD 0.68), with Groups 2, 3 and 4 also improving significantly (mean change 0.34 (SD 0.51), 0.34 (SD 0.61) and 0.71 (SD 0.78)) respectively (Table 3). Group 5 did not improve during follow-up (mean change 0.13, SD 0.66), and only 42% improved more than the minimal clinically important difference.

**Table 3:** Mean Change and Proportion Exceeding the Minimal Clinically important Difference in Physical Function, Fatigue, Physical Component Score, and Mental Component Score at 12 months by Trajectory Group*.

Group	1	2	3	4	5
	HDAS to REM	MDAS to REM	MDAS to LDAS	HDAS to LDAS	HDAS to MDAS
Mean (SD) Change in HAQ Score	-1.08 (0.68)	-0.34 (0.51)	-0.34 (0.61)	-0.71 (0.78)	-0.13 (0.66)
Proportion with HAQ Score Improving >0.22 units	206 (91%)	129 (52%)	207 (59%)	172 (76%)	51 (42%)
Mean (SD) Change in PCS	15.4 (10.1)	6.9 (10.3)	6.0 (10.3)	9.1 (12.1)	3.6 (10.4)
Proportion with PCS Score Improving >2.5 units	187 (91%)	136 (62%)	197 (63%)	135 (71%)	54 (51%)
Mean (SD) Change in MCS	8.2 (11.6)	3.1 (9.9)	1.8 (11.4)	5.0 (12.0)	2.6 (12.0)
Proportion with MCS Score Improving >2.5 units	136 (66%)	111 (51%)	143 (45%)	106 (56%)	52 (49%)
Mean (SD) Change in Fatigue (0–10 Visual Analogue Scale)	-4.1 (3.3)	-1.6 (3.1)	-1.2 (3.0)	-2.3 (3.3)	-1.0 (3.1)
Proportion with Fatigue Score Improving >2 units	180 (78%)	118 (47%)	147 (41%)	136 (57%)	50 (40%)

* For all Comparisons p value<0.001. HDAS, high disease activity state (DAS28 > 5.1); REM, remission (DAS28 <2.6); MDAS, moderate disease activity state (DAS28 3.2–5.1); LDAS, low disease activity state (DAS28 2.6–3.2)

### Quality of Life Measures

Baseline values by trajectory group are reported in Table 2. Groups 1, 4 and 5 have the worst baseline mean Physical Component Score, with Group 1 improving by a mean of 16.4 (SD 10.2) units compared to 9.9 (SD 12.0) units in Group 4 and 3.8 (SD 10.5) units in Group 5 in the first 12 months (between group differences p<0.001, Table 3). Group 1 also has the greatest improvement in mean MCS at 9.7 (SD 12.5) units compared to Group 4 with 5.5 (SD 12.9) units, relative to improvements in Groups 2, 3 and 5 ranging from 2.3–3.6 units (between group differences p<0.001). Group 1 and Group 4 were the only groups to have a significant improvement in fatigue (>2 units) over 12 months (Group 1–4.1 (SD 3.3) units, Group 4–2.3 (SD 3.3) units, compared to -1.0 to -1.6 in other groups; between group differences p<0.001).

## Discussion

Our study uses group-based trajectory modelling to demonstrate distinct heterogeneity in the course of disease in ERA, which is ultimately reflected in variable degrees of radiographic damage progression and improvements in physical function and quality of life measures. We detected five discrete disease trajectories, categorized by the disease activity state at inception to the cohort and then observed over two years. These trajectories are characterized by variations in sociodemographic and health status factors at baseline and less so disease characteristics, and which emerge despite the initial aggressive treatment received. Patients in the group experiencing the largest improvement in function and quality of life measures (Group 1, HDAS to REM) and the group with the best prognosis (Group 2, MDAS to REM, with the least radiographic progression and treatment needs) are more often <50 years old, with less medical comorbidity. Patients with the worst prognosis (Group 4, beginning in HDAS and achieving LDAS with high odds for radiographic progression; Group 5 beginning in HDAS and only achieving MDAS) are more likely to have lower levels of education, are less frequently employed and have lower total household incomes. Group 5 is additionally characterized by a high proportion of non-Caucasian ethnicities. These findings speak to the importance of addressing social determinants of health to improve ERA outcomes, as even aggressive treatment could not prevent radiographic progression nor provide robust improvements in quality of life measures.

Although the relationship of treatment with these trajectories is complicated, the majority of patients were treated aggressively with methotrexate-based regimens in doses exceeding 19 mg weekly. The group with the worst disease activity trajectory (Group 5) had less frequent use of methotrexate (albeit in similar doses if used compared to the other groups) and other DMARDs and more corticosteroid and biologic use during follow-up, without realizing the treatment goal of LDAS or REM. This group may represent patients who have had contraindications to usual first-line therapies, or differences in tolerating, adhering to, or accepting use of DMARD medication at higher doses, possibly influenced by cultural differences or health literacy [17]. Innate heterogeneous biologic differences are also likely to contribute to disease course, reflected in the very different responses between groups 1 and 4, in which methotrexate therapy is used similarly. To illustrate, Groups 1 and 4 both started in high disease activity and received similar treatment, yet only patients in Group 1 were rapid responders to methotrexate (DAS28 reduction of 2.96 within 3 months). Given these observed differences in treatment response, there is an opportunity to identify unique molecular signatures in these discrete groups that could inform patient-specific initial therapeutic choices.

Our study complements the study by Siemons et al [3] in which trajectories of ‘Fast Response’, ‘Slow Response’ and ‘Poor Outcome’ groups were identified over 12 months despite a treat to target strategy, although notably with lower doses of methotrexate used compared to the population we describe. That study compared baseline characteristics for disease activity (DAS28, tender and swollen joint counts, inflammatory markers), disease characteristics (rheumatoid factor positive), limited sociodemographic factors (body mass index, age, sex), and selected patient-reported and well-being measures (patient global, pain, SF-36 physical, SF-36 mental) and only male sex was predictive of fast response. They concluded that future studies should attempt to identify more specific risk factors for poor outcome, to enable early identification of patients in need of alternative therapeutic approaches. Norton has examined heterogeneity in ERA by defining functional trajectories using scores from the Health Assessment Questionnaire, and examining the relationship with mortality [18] and disability progression [19]. Norton also described sociodemographic differences between trajectory groups, notably that females, higher medical comorbidity scores, low education, low social class and low employment levels predominate in the group with the highest level of HAQ disability. This group also experienced higher mortality after adjustment for important confounders. Although our study did not use a mortality endpoint, we identified that significant radiographic progression, which is tightly associated with disability [20], was observed in one-third of patients in the groups with HDAS at treatment initiation and a flat response curve, in just one year of observation. Thus, we might expect to see findings similar to that of Norton in our cohort in the long-term. Taken together, these data demonstrating disease heterogeneity underscore the need for early identification of patient-specific factors to predict a patient’s destined trajectory.

The GBTM strategy had its origins in criminology theory, but has gained a foothold in psychology, mental health, epidemiology, chronic disease and medication adherence studies [21, 22]. The advantages of this approach in longitudinal data analysis are the ability to statistically derive whether true heterogeneity exists in a dataset, while accounting for possible chance differences across individuals [23]. Patterns of outcomes are identified, and the selection of the number of trajectories are informed statistically, thus providing more objective analysis rather than just applying a simple dichotomous outcome selected by the researcher. As an example of application from the modelling to the clinical setting, a recent Canadian retrospective study of swollen joint count trajectories in juvenile inflammatory arthritis was performed [24]. Berard et al. identified five latent classes, which were clinically and statistically distinct from the International League of Associations for Rheumatology (ILAR) categorizations. Thus this type of analytic approach will allow us to redefine outcome trajectories that reflect heterogeneity within diseases. An additional advantage of this modelling strategy is that it allows analysis of baseline predictors for heterogeneous outcomes, thus providing a structure to explore personalized treatment selection.

Limitations of the study were addressed where possible. Longitudinal studies are susceptible to bias from patients lost to follow-up, who may differ systematically from patients remaining in the study. To assess the potential impact of incomplete follow-up, we performed the analysis on 343 patients with complete 2-year follow-up data. The model continued to identify 5 trajectories with proportionately similar numbers of patients in each group, providing further credence to the observed heterogeneity in this population. Similarly, when we performed the analysis on treatment naïve subjects or those minimally exposed to methotrexate our results did not change (S1 Fig). Moreover, this analytical method allows inclusion of all data when missing data are at random. Employment status at entry to the cohort may already reflect consequences of the disease rather than a sociodemographic factor that influences prognosis. Additionally, unmeasured confounders associated with disease trajectories such as therapeutic intolerances and adherence may not be evenly distributed across groups.

## Conclusion

In the era of cost constraints, means to identify patients destined to have a severe disease course using novel statistical methods, additional variables, or biomarkers are desired, which will allow provision of the right therapy for the right patient resulting in maximized function, gainful employment and less destruction from this disease. These data confirm that heterogeneous disease trajectories exist in ERA, and that emerge despite initial aggressive therapy. This may reflect limitations in the use of available therapies due to patient comorbidities, health literacy, or individual response to standard therapies.

## Supporting Information

S1 Fig Predicted Group Trajectories in Treatment Naïve or Minimally Exposed Early Rheumatoid Arthritis based on DAS28 with 95% CI (n = 343). Five predicted group trajectories (solid or dashed lines) and 95% confidence interval limits (shaded) are depicted from the group-based trajectory modelling. Percentages reflect the predicted proportion of subjects in each group, which differs marginally from the actual group characterization in the dataset.
(TIFF)

## References

[1] van Riel PL, van Gestel AM. Clinical outcome measures in rheumatoid arthritis. Ann Rheum Dis. 2000;59 Suppl 1:i28–31. 11053082 10.1136/ard.59.suppl_1.i28 PMC1766622

[2] Aletaha D, Funovits J, Keystone EC, Smolen JS. Disease activity early in the course of treatment predicts response to therapy after one year in rheumatoid arthritis patients. Arthritis Rheum. 2007;56(10):3226–35. 17907167 10.1002/art.22943

[3] Siemons L, Ten Klooster PM, Vonkeman HE, Glas CA, Van de Laar M. Distinct trajectories of disease activity over the first year in early rheumatoid arthritis patients following a treat-to-target strategy. Arthritis Care Res (Hoboken). 2014;66(4):625–30. 24106173 10.1002/acr.22175

[4] Bykerk VP, Jamal S, Boire G, Hitchon CA, Haraoui B, Pope JE, et al The Canadian Early Arthritis Cohort (CATCH): patients with new-onset synovitis meeting the 2010 ACR/EULAR classification criteria but not the 1987 ACR classification criteria present with less severe disease activity. J Rheumatol. 2012;39(11):2071–80. 10.3899/jrheum.120029 22896026

[5] Aletaha D, Neogi T, Silman AJ, Funovits J, Felson DT, Bingham CO 3rd, et al 2010 Rheumatoid arthritis classification criteria: an American College of Rheumatology/European League Against Rheumatism collaborative initiative. Arthritis Rheum. 2010;62(9):2569–81. 10.1002/art.27584 20872595

[6] Arnett FC, Edworthy SM, Bloch DA, McShane DJ, Fries JF, Cooper NS, et al The American Rheumatism Association 1987 revised criteria for the classification of rheumatoid arthritis. Arthritis Rheum. 1988;31(3):315–24. 3358796 10.1002/art.1780310302

[7] Bruce B, Fries JF. The Health Assessment Questionnaire (HAQ). Clin Exp Rheumatol. 2005;23(5 Suppl 39):S14–8. 16273780

[8] Prevoo ML, van 't Hof MA, Kuper HH, van Leeuwen MA, van de Putte LB, van Riel PL. Modified disease activity scores that include twenty-eight-joint counts. Development and validation in a prospective longitudinal study of patients with rheumatoid arthritis. Arthritis Rheum. 1995;38(1):44–8. 7818570 10.1002/art.1780380107

[9] van der Heijde D. How to read radiographs according to the Sharp/van der Heijde method. J Rheumatol. 1999;26(3):743–5. 10090194

[10] Bruynesteyn K, van der Heijde D, Boers M, Saudan A, Peloso P, Paulus H, et al Determination of the minimal clinically important difference in rheumatoid arthritis joint damage of the Sharp/van der Heijde and Larsen/Scott scoring methods by clinical experts and comparison with the smallest detectable difference. Arthritis Rheum. 2002;46(4):913–20. 11953967 10.1002/art.10190

[11] Iqbal SU, Rogers W, Selim A, Qian S, Lee A, Ren XS, et al. The Veterans RAND 12 Item Health Survey (VR-12): What It Is and How It Is Used. Technical Report. 2007.

[12] Lubeck DP. Patient-reported outcomes and their role in the assessment of rheumatoid arthritis. Pharmacoeconomics. 2004;22(2 Suppl 1):27–38. 15157002 10.2165/00019053-200422001-00004

[13] Pouchot J, Kherani RB, Brant R, Lacaille D, Lehman AJ, Ensworth S, et al Determination of the minimal clinically important difference for seven fatigue measures in rheumatoid arthritis. J Clin Epidemiol. 2008;61(7):705–13. 10.1016/j.jclinepi.2007.08.016 18359189 PMC2486378

[14] Nagin D. Group-based modeling of development Cambridge: Harvard University; 2005.

[15] Jones BL, Nagin DS, Roeder K. A SAS Procedure Based on Mixture Models for Estimating Developmental Trajectories. Sociological Methods & Research. 2001;29(3):374–93.

[16] Baron G, Ravaud P, Samson A, Giraudeau B. Missing data in randomized controlled trials of rheumatoid arthritis with radiographic outcomes: a simulation study. Arthritis Rheum. 2008;59(1):25–31. 10.1002/art.23253 18163406

[17] Quinlan P, Price KO, Magid SK, Lyman S, Mandl LA, Stone PW. The Relationship Among Health Literacy, Health Knowledge, and Adherence to Treatment in Patients with Rheumatoid Arthritis. HSS Journal. 2013;9:42–9. 10.1007/s11420-012-9308-6 24426844 PMC3640723

[18] Norton S, Sacker A, Dixey J, Done J, Williams P, Young A, et al Trajectories of functional limitation in early rheumatoid arthritis and their association with mortality. Rheumatology (Oxford). 2013;52(11):2016–24. 23934221 10.1093/rheumatology/ket253

[19] Norton S, Fu B, Scott DL, Deighton C, Symmons DP, Wailoo AJ, et al Health Assessment Questionnaire disability progression in early rheumatoid arthritis: systematic review and analysis of two inception cohorts. Semin Arthritis Rheum. 2014;44(2):131–44. 10.1016/j.semarthrit.2014.05.003 24925692 PMC4282305

[20] Bombardier C, Barbieri M, Parthan A, Zack DJ, Walker V, Macarios D, et al The relationship between joint damage and functional disability in rheumatoid arthritis: a systematic review. Ann Rheum Dis. 2012;71(6):836–44. 10.1136/annrheumdis-2011-200343 22128079

[21] Nagin DS, Odgers CL. Group-Based Trajectory Modeling (Nearly) Two Decades Later. Journal of quantitative criminology. 2010;26(4):445–53. 21132047 10.1007/s10940-010-9113-7 PMC2994902

[22] Franklin JM, Shrank WH, Pakes J, Sanfelix-Gimeno G, Matlin OS, Brennan TA, et al Group-based trajectory models: a new approach to classifying and predicting long-term medication adherence. Med Care. 2013;51(9):789–96. 10.1097/MLR.0b013e3182984c1f 23685406

[23] Nagin DS, Odgers CL. Group-based trajectory modeling in clinical research. Annual review of clinical psychology. 2010;6:109–38. 10.1146/annurev.clinpsy.121208.131413 20192788

[24] Berard RA, Tomlinson G, Li X, Oen K, Rosenberg AM, Feldman BM, et al Description of Active Joint Count Trajectories in Juvenile Idiopathic Arthritis. J Rheumatol. 2014;41:2466–73. 10.3899/jrheum.130835 25274882

