# Supplementary figures and images for: Heterogeneous Disease Trajectories Explain Variable Radiographic, Function and Quality of Life Outcomes in the Canadian Early Arthritis Cohort (CATCH)
Source: PLoS One. 2015 Aug 24;10(8):e0135327. doi: 10.1371/journal.pone.0135327 (PMC4547697; doi:10.1371/journal.pone.0135327)

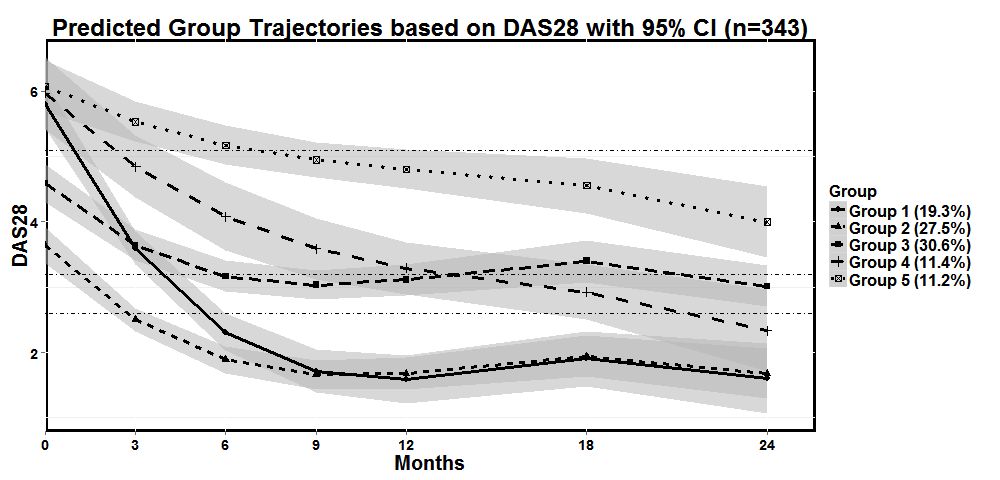

Supplement: S1 Fig — Five predicted group trajectories (solid or dashed lines) and 95% confidence interval limits (shaded) are depicted from the group-based trajectory modelling. Percentages reflect the predicted proportion of subjects in each group, which differs marginally from the actual group characterization in the dataset. (TIFF) [file pone.0135327.s001.tiff]
